# Rhabdomyolysis Following Rapid Correction of Acute Hyponatraemia in a Patient With Psychogenic Polydipsia

**DOI:** 10.7759/cureus.116201

**Published:** 2026-09-14

**Authors:** Annabelle Prosser, Qazi Imtiazuddin

**Affiliations:** 1 Internal Medicine, University College London Hospitals NHS Foundation Trust, London, GBR; 2 Endocrinology, The Newcastle upon Tyne Hospitals NHS Foundation Trust, Newcastle upon Tyne, GBR

**Keywords:** creatine kinase, hyponatraemia, psychogenic polydipsia, rapid hyponatraemia correction, rhabdomyolysis

## Abstract

Rhabdomyolysis is a recognised but under-reported complication of acute hyponatraemia and its treatment, particularly in patients with psychogenic polydipsia. We report a case of a male in his 50s with recurrent psychogenic polydipsia who developed severe rhabdomyolysis as a potential result of both hyponatraemia and its subsequent rapid correction. Serum sodium was 117 mmol/L on admission and increased to 131 mmol/L within six hours on venous blood gas sampling following administration of hypertonic saline. Creatine kinase (CK) was already elevated at admission and rose sharply following rapid administration of hypertonic saline, peaking at 60,672 U/L, with preserved renal function throughout admission. Alternative causes of rhabdomyolysis were explored and systematically excluded. This case highlights the importance of cautious sodium correction, continued biochemical monitoring beyond initial stabilisation, and consideration of CK surveillance in high-risk patients.

## Introduction

Psychogenic polydipsia is most commonly described in patients with chronic psychiatric illness and is a recognised cause of acute dilutional hyponatraemia. This can result in severe neurological sequelae such as headache, seizures, altered consciousness, and death [1].

Up to 25% of patients with schizophrenia have been reported to experience psychogenic polydipsia. However, establishing an exact prevalence of hyponatraemia remains challenging. This is because patients often experience gradual, asymptomatic reductions in serum sodium, which can precipitate acute hyponatraemic crises [2].

While complications of hyponatraemia overcorrection, such as osmotic demyelination, are well described [3], rhabdomyolysis is a less frequently recognised association [4], with very few previously documented cases linking it specifically to psychogenic polydipsia and its subsequent correction [5,6]. Rapid sodium shifts can compound this damage by inducing acute osmotic stress in vulnerable myocytes [5]. Rhabdomyolysis is characterised by skeletal myocyte lysis causing the release of intracellular components such as electrolytes, myoglobin, and creatine kinase (CK) into the circulation. The most common causes include trauma and toxins; however, electrolyte disturbances are a recognised trigger [7]. Furthermore, a growing body of literature recognises that profound hyponatraemia itself, independent of its correction, can directly precipitate rhabdomyolysis [8-10].

We present a case of severe rhabdomyolysis following the rapid correction of acute hyponatraemia in a patient with psychogenic polydipsia. This case adds to the literature by illustrating a potential link between rapid sodium correction and sequentially compounding myocyte injury, whilst acknowledging the contribution of hyponatraemia itself alongside other factors.

## Case presentation

A male in his 50s presented following a fall preceded by dizziness, with no associated long lie. He had a known history of psychogenic polydipsia diagnosed in 2022, with a previous admission in 2023 for hyponatraemia, dropping to 115 mmol/L at its lowest. Notably, CK had not been measured during that prior episode. His sodium normalised prior to this admission. His other past medical history included depression and erosive gastritis. On admission, his medications included amitriptyline hydrochloride 10 mg at night, risperidone 2 mg at night, and a nicotine replacement patch. He had no recent medication changes and reported compliance. He reported no alcohol or recreational drug use. He was a smoker of roughly 20 cigarettes a day. The patient lived in sheltered accommodation and received twice-daily carer visits. Collateral history was obtained from a carer who heard the fall, providing an estimate of less than a minute on the floor and denied witnessing any seizure activity. The patient reported no muscle pain, weakness, or agitation, undertook no physical activity before the fall, and experienced no recent infections.

The patient was asymptomatic and mobilising independently in the ward. A full physical examination revealed no obvious pain, bruising, or haematomas of the body. He was clinically euvolaemic. A detailed neurological examination, including assessment of cranial nerves, tone, power, coordination, reflexes, and gait, was entirely normal with no focal deficits, extrapyramidal signs, or altered mental status.

Investigations

Admission blood tests demonstrated acute hyponatraemia with mild hypokalaemia. Serum sodium was 117 mmol/L. Key biochemical trends during admission, including serum sodium, CK, and renal function, are summarised in Table 1.

**Table 1:** Trends of laboratory investigations by day. Abbreviations: CK, creatine kinase; CRP, C-reactive protein; TSH, thyroid-stimulating hormone. Values denoted by "–" were not clinically indicated for daily serial measurement. Daily serial monitoring of CK, electrolytes, and renal function was prioritised to track the clinical trajectory and recovery of severe rhabdomyolysis. Day 1 marks initial hospital admission. ^a ^Urinary sodium and urine osmolality were selectively measured on day 2 to evaluate the underlying aetiology of acute hyponatraemia. ^b ^Cortisol and TSH were assessed on initial presentation to rule out secondary endocrine aetiologies (hypothyroidism and adrenal insufficiency). Reference ranges: sodium, 133–146 mmol/L; potassium, 3.5–5.3 mmol/L; creatinine, 59–104 µmol/L; CRP, 0–4.9 mg/L; CK, 40–320 U/L; urinary sodium, variable (dependent on intake and hydration); urine osmolality, 50–1200 mmol/kg; cortisol, 140–690 nmol/L; TSH, 0.27–4.2 mIU/L.

Day	Sodium (mmol/L)	Potassium (mmol/L)	Creatinine (µmol/L)	CRP (mg/L)	CK (U/L)	Urinary sodium (mmol/L)^a^	Urine osmolality (mmol/kg)^a^	Cortisol (nmol/L)^b^	TSH (mIU/L)^b^
1	117	3.2	57	1.7	3,188	–	–	199	0.46
2	137	3.6	55	1.4	38,959	21	74	444	–
3	142	3.8	60	0.9	60,672	–	–	–	–
4	136	3.9	65	–	49,496	–	–	–	–
5	136	4.0	68	–	26,698	–	–	–	–
6	127	3.9	70	–	11,377	–	–	–	–
7	133	4.1	72	–	5,514	–	–	–	–
8	133	4.0	74	–	3,534	–	–	–	–
9	139	4.2	76	–	1,971	–	–	–	–
13	138	–	–	–	822	–	–	–	–

Alternative causes of hyponatraemia were appropriately investigated, as detailed in Table 1. Thyroid function tests and cortisol were within normal limits, excluding hypothyroidism and adrenal insufficiency, while the low urinary sodium and urine osmolality were consistent with a dilutional hyponatraemia, in keeping with psychogenic polydipsia.

Differential diagnosis

Differential causes of the rapid rise in CK were systematically excluded after advice was sought from orthopaedic, psychiatry, and rheumatology teams. Orthopaedic review found no evidence of compartment syndrome. The psychiatry team considered medication-induced CK rise to be unlikely, such as from his risperidone or amitriptyline use. Nevertheless, both agents were temporarily withheld during the admission to assess for any improvement. He had been on both of these medications for two years with no recent dose changes. He reported no symptoms suggestive of an inflammatory myopathy (e.g., proximal muscle weakness and rash), making a primary rheumatological cause of CK elevation unlikely; therefore, the rheumatology team did not recommend further investigation. Inflammatory markers remained low throughout admission with no symptoms suggestive of a systemic infection.

Management

Given his acute hyponatraemia, the patient was admitted to a monitored medical unit. He received treatment following admission bloods on day 1 consisting of three 150 mL boluses of hypertonic saline (2.7% sodium chloride) administered over 20 minutes each, spaced across the first six hours of treatment. Of note, serial sodium measurements in between these individual boluses were not performed.

Repeat venous blood gas sampling demonstrated an increase in serum sodium from 117 mmol/L before hypertonic saline administration to 131 mmol/L after treatment six hours later, representing rapid correction [3,11], a trend subsequently confirmed by formal laboratory biochemistry on day 2. Given this rapid rise, further hypertonic saline administration was immediately withheld. Specific active re-lowering interventions (such as desmopressin or hypotonic fluids) were not administered, as the patient remained entirely asymptomatic and neurologically stable.

The renal team was consulted to support ongoing monitoring and optimisation of renal function. They advised initiation of sodium bicarbonate 500 mg twice daily, alongside ongoing hydration as his hyponatraemia settled.

Outcome and follow-up

CK peaked at 60,672 U/L and subsequently declined with preserved renal function. His sodium level was 138 mmol/L on discharge, within the normal range. The patient had no immediate complications and was deemed medically fit for discharge after 10 days of admission, with no neurological symptoms. He was followed up by the community mental health team.

## Discussion

In this case, serum sodium increased by 14 mmol/L within six hours, exceeding guidance on recommended correction thresholds [3,11]. Crucially, the absence of serial sodium checks between the individual hypertonic saline boluses likely contributed to inadvertent overcorrection. Current guidelines for the management of acute hyponatraemia emphasise controlled correction, highlighting that there is no evidence that correcting by >6 mmol/L within 24 hours improves outcomes in acute or chronic hyponatraemia. However, exact correction targets depend on clinical context, chronicity, and risk profile of the patient [11].

As demonstrated in Table 1, a critical finding in our data is the baseline CK elevation of 3,188 U/L recorded on admission (day 1), prior to the administration of hypertonic saline. This indicates that myocyte injury had already occurred at presentation. Given that this initial elevation occurred prior to the rapid sodium correction, this could implicate profound acute hyponatraemia itself (117 mmol/L) as the trigger for osmotic cellular stress and membrane instability. In addition, the subsequent rapid sodium correction temporally correlates with a sudden escalation in muscle breakdown, driving CK levels to a peak of 60,672 U/L. This raises the possibility that rapid sodium correction may have accelerated rhabdomyolysis, as described in previous literature, which identifies it as a key risk factor for myocyte injury [12]. Thus, the clinical timeline could point towards a compounding effect: hyponatraemia initiated the myocyte injury, which was subsequently exacerbated by rapid osmotic shifts during correction. Alternative causes of rhabdomyolysis were carefully excluded, including trauma, prolonged immobility, medication-induced myotoxicity, and inflammatory myopathies, thereby strengthening this association.

Given his rapid sodium correction, he was at theoretical high risk for osmotic demyelination syndrome (ODS) [3]. However, despite exceeding these thresholds, the patient remained neurologically intact, alert, and free of neurological deficits, movement disorders, or altered mental status throughout his hospital stay. Consequently, further imaging such as magnetic resonance imaging (MRI) was not performed.

The pathophysiology of rhabdomyolysis in hyponatraemia is not fully understood but is hypothesised to involve osmotic shifts leading to myocyte swelling, membrane instability, and subsequent cell lysis. Another mechanism postulated is the interference with the Na+/Ca++ exchanger. The reduction of the Na+ gradient causes a reduction in Ca++ efflux, thus producing a higher level of intracellular Ca++ to accelerate the cellular death process [4].

The patient's continuous use of nicotine patches represents a potential, albeit minor, cofactor. There is some limited evidence that prolonged nicotine exposure may have heightened myocyte susceptibility to osmotic stress [13].

Despite this profound CK elevation, renal function remained preserved throughout the admission. This clinical course likely resulted from prompt hydration and sodium bicarbonate therapy, the absence of concurrent nephrotoxins or sepsis, as well as the transient nature of the peak.

Quantitative urinary myoglobin was not measured during admission as it has a limited clinical utility due to its short half-life (two to three hours) and high rate of false-negative results [14]. The diagnosis of severe rhabdomyolysis was established by the marked elevation in CK.

Following initial stabilisation, the patient experienced a secondary drop in serum sodium to 127 mmol/L on day 6 (Table 1). This fluctuation was not a deliberate therapeutic re-lowering intervention but rather reflected a breakthrough relapse of psychogenic polydipsia driven by covert water consumption in the ward, highlighting the persistent difficulties of maintaining fluid restriction despite a supervised hospital environment.

Limitations

This case report has several inherent limitations. Firstly, as a single-patient case report, these observations demonstrate a clinically plausible temporal association rather than definitive causality and lack a control group to quantify relative risk or incidence. Secondly, the precise individual contributions of profound hypotonicity, rapid overcorrection, and potential cofactors such as psychogenic polydipsia, the fall itself, and nicotine patch use cannot be fully isolated or quantified.

Additionally, whilst skeletal muscle breakdown was severe, troponin testing was not performed during admission due to the absence of cardiac symptoms or haemodynamic instability. An electrocardiogram performed on presentation demonstrated normal sinus rhythm, without acute ischaemic changes, arrhythmias, or corrected QT (QTc) prolongation. Nonetheless, subclinical myocardial injury cannot be definitively excluded in the absence of routine cardiac enzyme surveillance. Furthermore, a routine urine dipstick was not performed during the acute presentation, precluding the assessment of haem positivity as a supportive clinical marker. Due to the absence of serial sodium measurements between individual hypertonic saline boluses, the exact trajectory of sodium correction within the six-hour window remains unknown. Consequently, the precise kinetics of correction cannot be definitively established beyond our available venous blood gas and laboratory values. Future prospective studies would be valuable in delineating the independent impact of rapid sodium overcorrection on the development of rhabdomyolysis.

## Conclusions

Rhabdomyolysis represents an important but under-recognised complication of both acute hyponatraemia and its treatment. This case suggests a temporal association of profound hyponatraemia and rapid sodium shifts with CK elevation. This phenomenon occurred in the absence of neurological symptoms or acute kidney injury, highlighting that rhabdomyolysis can remain clinically silent.

Given these risks, this case underscores the importance of strictly adhering to recommended sodium correction thresholds through frequent serial sodium measurements between hypertonic saline boluses to prevent overcorrection. Furthermore, in selected high-risk patients presenting with severe hyponatraemia and psychogenic polydipsia, targeted CK monitoring merits consideration, especially when muscle injury is potentially compounded by rapid correction. Further research is needed to investigate the individual contributions of hyponatraemia versus its correction to the development of rhabdomyolysis. Broadening awareness of this complication will facilitate earlier recognition and improve management in similar clinical presentations.
